# Supplementary material for: IL-15 and PIM kinases direct the metabolic programming of intestinal intraepithelial lymphocytes
Source: Nat Commun. 2021 Jul 13;12:4290. doi: 10.1038/s41467-021-24473-2 (PMC8277781; doi:10.1038/s41467-021-24473-2)
Supplement: Supplementary file 1 — Supplementary Information [file 41467_2021_24473_MOESM1_ESM.pdf]

IL-15 and PIM kinases direct the metabolic programming of intestinal intraepithelial lymphocytes

Olivia J. James, Maud Vandereyken, Julia M. Marchingo, Francois Singh, Susan E. Bray, Jamie Wilson, Andrew G. Love, Mahima Swamy

Supplementary Information

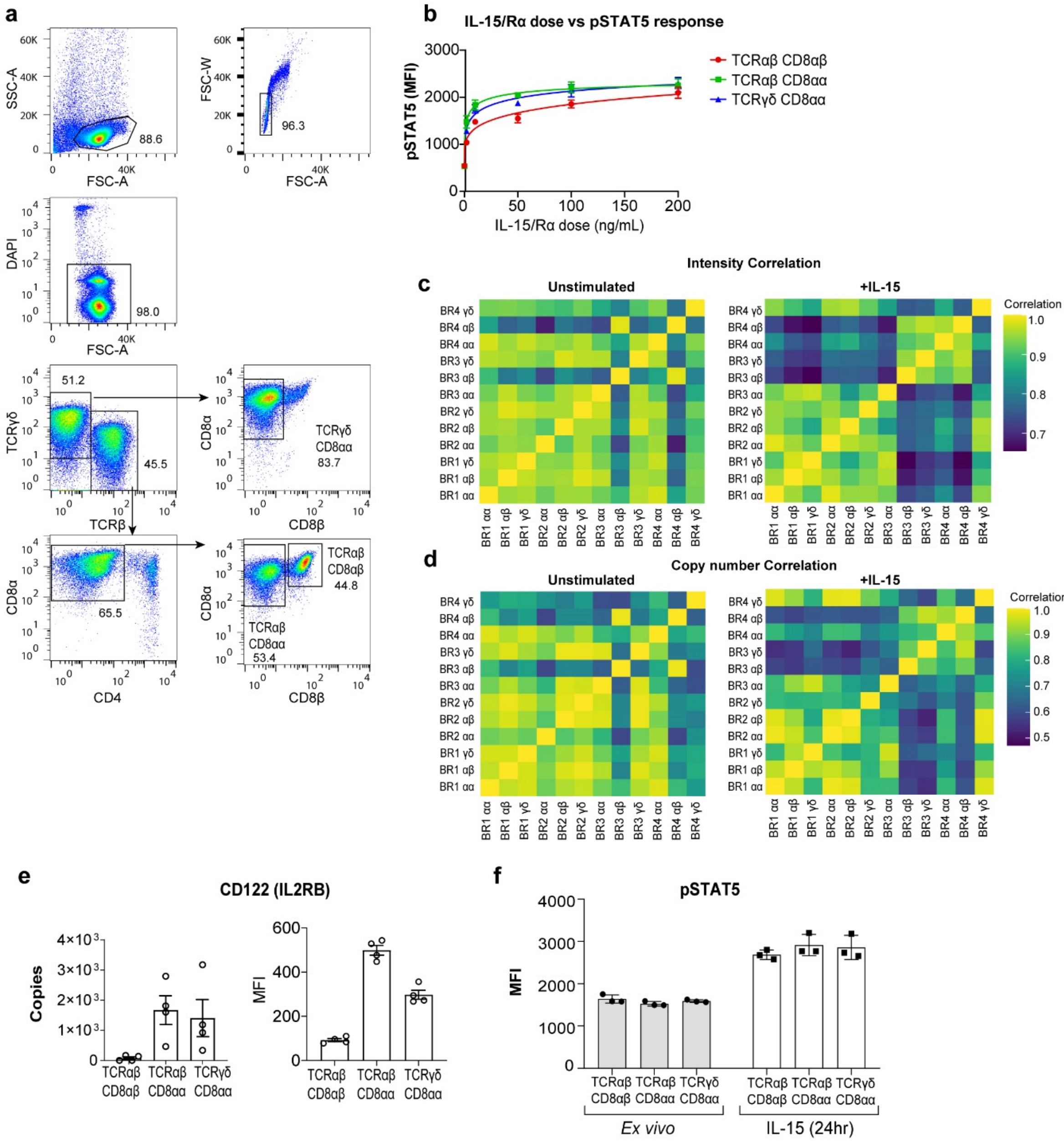

Supplementary Figure 1. Global analyses and correlations of IEL proteomes with and without IL-15 stimulation

(**a**) The gating strategy used to identify and isolate IEL subpopulations for fluorescence activated cell sorting (FACS). Cells were first gated on size using forward scatter (FSC) vs side scatter (SSC) to separate out cells of interest from debris/non-lymphoid cells, then FSC-Width to separate out single cells. DAPI-negative cells were considered live and were further separated into three distinct IEL subpopulations based on cell surface marker expression of T cell-associated receptors; TCRγδ, TCRβ, CD4, CD8α and CD8β. The populations sorted were as follows; those that were TCRγδ+ CD8αα+, those that were both TCRαβ+ CD4- *and* either CD8αα+ or CD8αβ+. (**b**) Phospho-STAT5 (Tyr694) response curve vs IL-15/Rα dose in IEL that were stimulated for 3hrs with increasing concentrations of IL-15/Rα (0ng/mL, 2ng/mL, 10ng/mL, 50ng/mL, 100ng/mL and 200ng/mL). Cells were collected and pSTAT5 levels were assessed by Fluorescent Cell Barcoding, data

is presented as mean fluorescence intensity (MFI) (n = 3 biologically independent experiments), and line graphs are fitted with non-linear regression analysis. (**c**) Heat maps show the Pearson's correlation coefficients of the raw intensities of all proteins identified in the data set for each IEL subset relative to each biological replicate. A heat map was generated for both the *ex vivo* samples (left) and the IL-15-treated samples (right) distinctively. BR = biological replicate (e.g. BR1 = Biological replicate 1). αβ = TCRαβCD8αβ. αα = TCRαβCD8αα. γδ = TCRγδCD8αα. (**d**) Protein intensities were converted into estimated copy numbers per cell and heatmaps were generated as in (c). (**e**) Estimated copy numbers/cell of the β-chain of the IL-15R (CD122/IL2RB) in each IEL subset (n = 4 biologically independent samples) and flow cytometric analysis of CD122 presented as MFI (gating strategy shown in Fig. S6c, n = 4 biologically independent experiments). (**f**) Flow cytometric analysis of pSTAT5 from *ex vivo* IEL and IEL treated with 100ng/mL IL-15/Rα for 24hrs (gating strategy shown in Fig. S6b, n = 3 biologically independent experiments). Data presented as mean fluorescence intensity (MFI). Error bars are mean ± s.e.m.

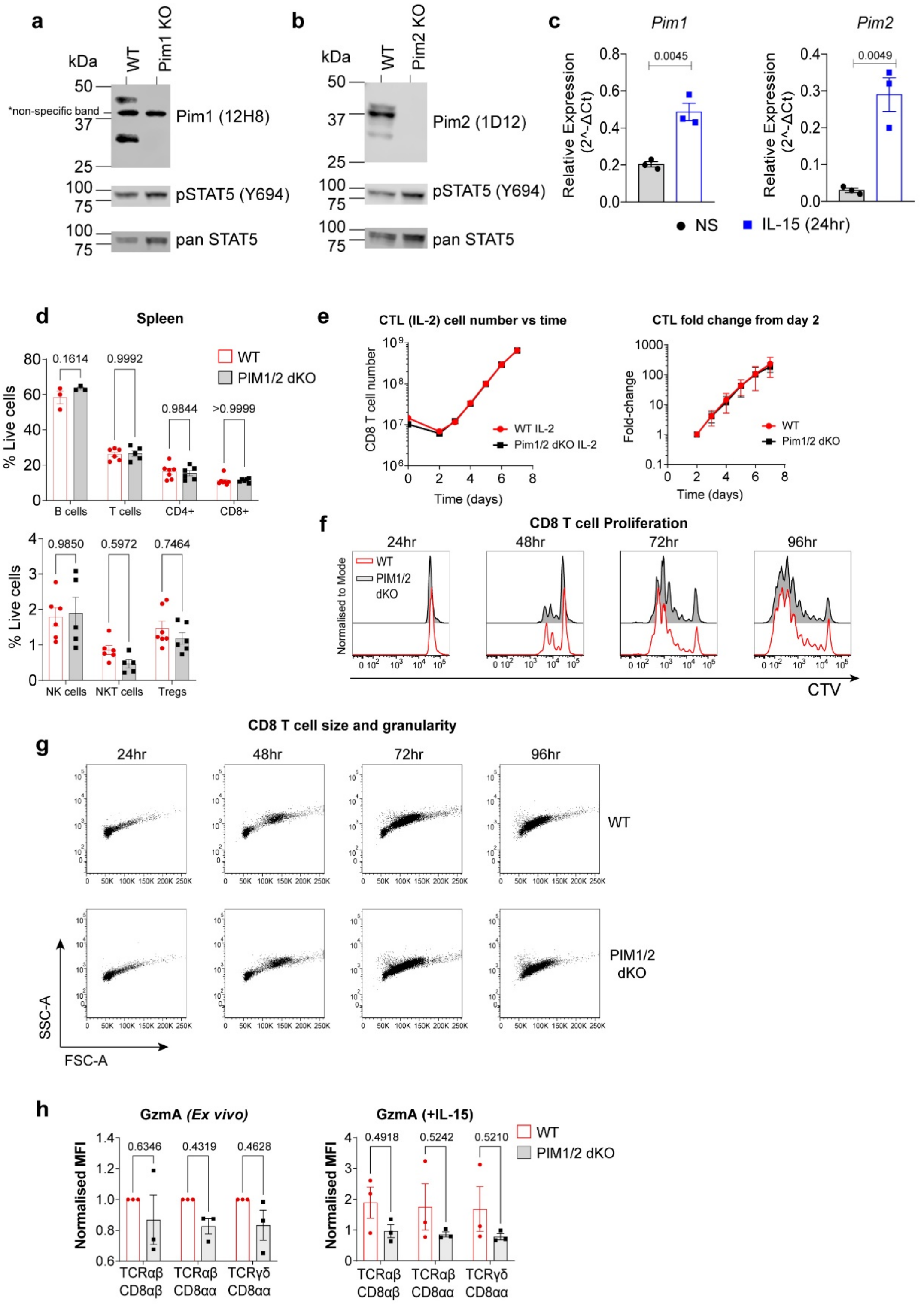

Supplementary Figure 2. Controls and analyses of splenocytes and IL-2-stimulated CTL from PIM1/2 dKO

(**a**) WT and *Pim1* KO Day 6 IL-2 expanded CTL were immunoblotted for PIM1 (12H8), pSTAT5 and panSTAT5. (**b**) WT and *Pim2* KO Day 6 IL-2 + IL-12 expanded CTL were immunoblotted for PIM2 (1D12), pSTAT5 and panSTAT5. (a) and (b) are n = 1 biologically independent experiment. (**c**) Relative expression of *Pim1* (left panel) and *Pim2* (right panel) mRNA in ex vivo IEL (grey) or IEL cultured with 100ng/mL IL-15/Rα for 24hrs

(blue) (n = 3 biologically independent samples). mRNA data were normalised to *Cd3ε.* Error bars are mean ± s.e.m. (**d**) Percentage of live cells from WT and PIM1/2 dKO spleens that were B cells ($B220^+TCRb^-$) (n = 3), T cells ($TCRb^+NK1.1^-$) (WT (n = 6) PIM1/2 dKO (n = 5), CD4 T cells ($TCRb^+NK1.1^-CD4^+$) (WT (n = 7) PIM1/2 dKO (n = 6), CD8 T cells ($TCRb^+NK1.1^-CD8^+$) (WT (n = 7) PIM1/2 dKO (n = 6), NK cells ($TCRb^-NK1.1^+$) (WT (n = 6) PIM1/2 dKO (n = 5), NKT cells ($NK1.1^+TCRb^+$) (WT (n = 6) PIM1/2 dKO (n = 5) or Tregs ($TCRb^+NK1.1^-CD4^+CD25^+$) (WT (n = 7) PIM1/2 dKO (n = 6). Symbols represent biological replicates; error bars are mean ± s.e.m. (**e**) CD8+ T cell number of WT and PIM1/2 dKO T cells was counted daily from day 2 of culture onwards (see methods for culture conditions). Left panel shows the cell number vs time (corrected for splitting). Data is representative of 4 independent experiments. Right panel shows the fold-change in cell number from day 2 over time. Data shows Mean ± SD (n = 7 biologically independent samples). (**f**) Lymph node single cell suspensions from WT and Pim1/2 dKO were labelled with cell trace violet (CTV), activated with anti-CD3 and anti-CD28 and CD8 T cell proliferation (CTV dilution) analysed by flow cytometry daily (gating strategy shown in Fig. S6e). (**g**) Corresponding cell size and granularity was measured by FSC-A and SSC-A, respectively. (f) and (g) Data shows representative plots from 2 independent experiments. (**h**) IEL from both WT and PIM1/2 dKO mice were isolated and stained for intracellular GzmA expression *ex vivo* (left) and following 96hrs in culture with 100ng/mL IL-15/Rα (right), gating strategy is shown in Fig. S1a, data presented as MFI normalised to WT controls (*ex vivo*) or to the respective WT or dKO IEL cultured in 2ng/mL IL-15/Rα (n = 3 biologically independent experiments). Error bars are mean ± s.e.m. Significance was calculated using two-way ANOVA and Sidak's multiple comparisons in (d) and (h), and two-tailed unpaired t-test for (c).

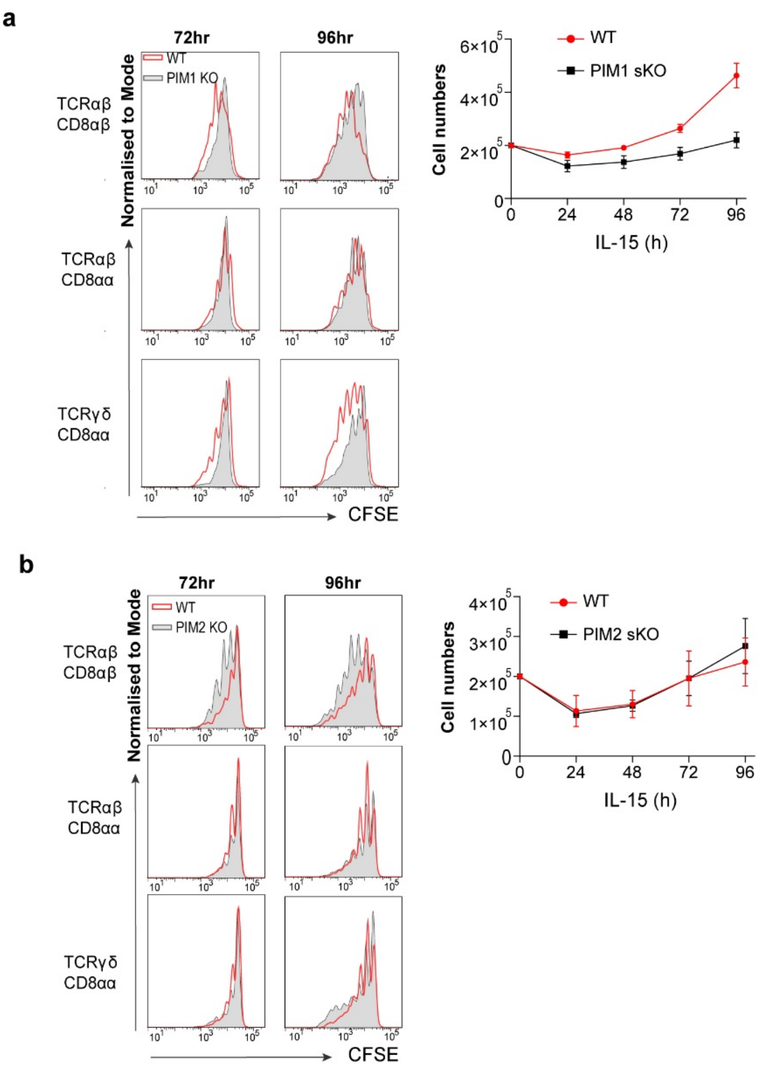

Supplementary Figure 3. IEL from *Pim1*$^{-/-}$ or *Pim2*$^{-/-}$ single KO (sKO) proliferate

IEL were stained with CellTrace CFSE prior to stimulation with 100ng/mL IL-15/Rα for 4 days. Every 24hrs cells were stained for IEL subsets (gating strategy as in Fig. S1a) and CFSE expression was analysed by flow cytometry. The discrete peaks in the histograms represent successive generations of live, DAPI-negative IEL for (**a**) PIM1 sKO (n = 3 biologically independent experiments) and (**b**) PIM2 sKO (n = 3 biologically independent experiments) IEL. Line graphs show the survival of IEL from either (**a**) PIM1 sKO (n = 4 biologically independent samples), or (**b**) PIM2 sKO (n = 4 biologically independent samples) mice as compared to IEL from wildtype mice that were cultured in 100ng/mL IL-15/Rα for 0-96hrs. Error bars are mean ± s.e.m.

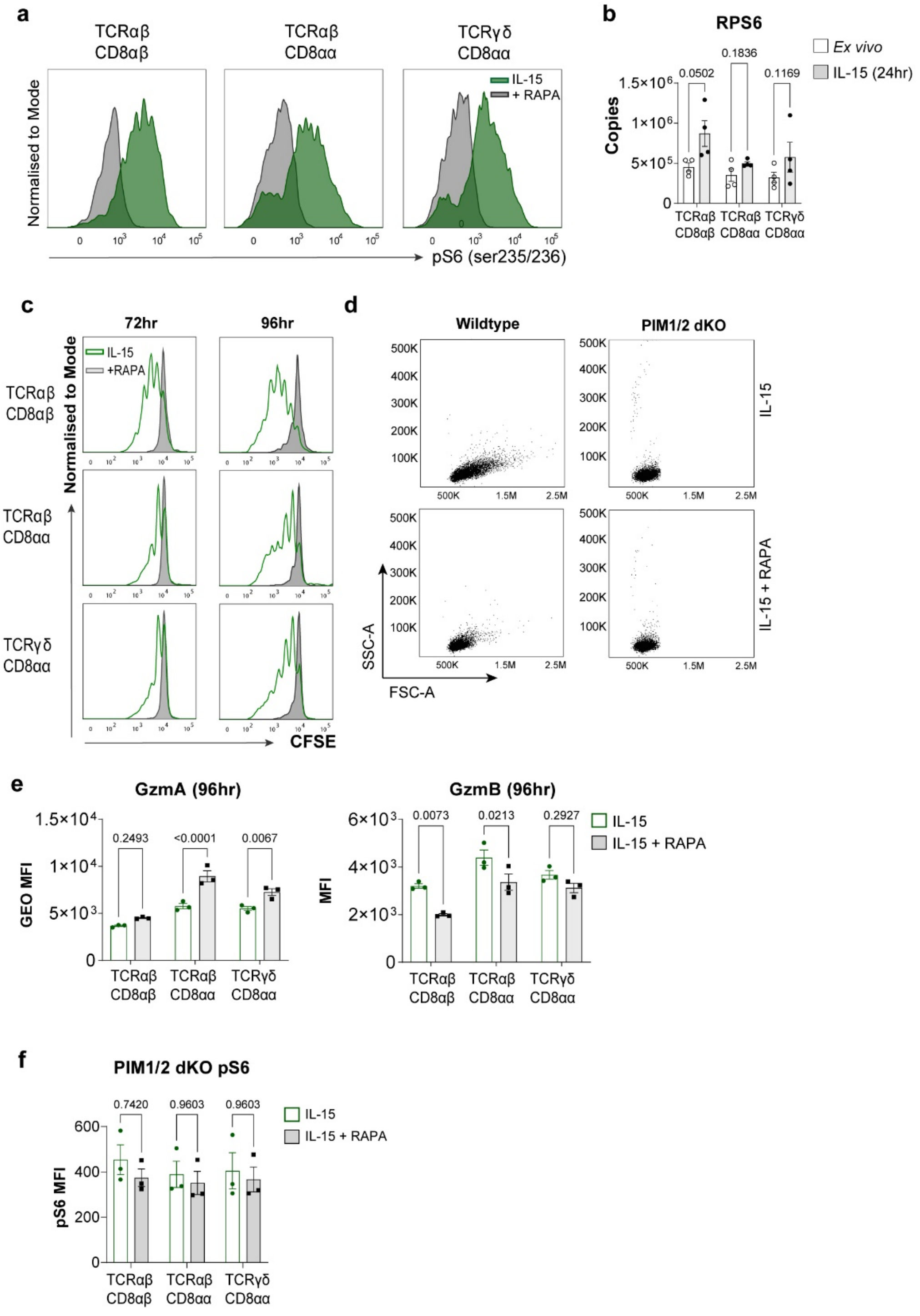

Supplementary Figure 4. Inhibition of mTORC1 blocks IL-15-induced proliferation and growth of IEL

(**a**) IEL were isolated and cultured in 100ng/ml IL-15/Rα with (grey) or without (green) 20nM rapamycin for 48hrs, histograms show phospho-S6 (Ser235/236) expression in IEL subsets (gating strategy shown in Fig. S6b) (representative of n = 4 biologically independent samples). (**b**) Estimated copy numbers/cell are shown for the Ribosomal Protein S6 (RPS6) in IEL subsets that were either untreated (*ex vivo*) or maintained in 100ng/mL IL-15/Rα for 24hrs (n = 4 biologically independent samples), statistical significance was derived from two-tailed empirical Bayes moderated *t*-statistics performed in limma on total proteome, see

Supplementary Data 3. (**c**) IEL were stained with CellTrace CFSE prior to stimulation with 100ng/mL IL-15/Rα (+/- 20nM rapamycin) for 4 days. At 72hrs and 96hrs cells were stained for subsets and CFSE expression was analysed by flow cytometry (gating strategy shown in Fig. S1a). The discrete peaks in the histograms represent successive generations of live, DAPI-negative IEL (representative of n = 3 biologically independent samples). (**d**) Dot plots show the forward scatter (FSC) vs side scatter (SSC) of live IEL from wildtype and PIM1/2 dKO mice cultured in 100ng/mL IL-15/Rα alone as compared to IEL cultured in 100ng/mL IL-15/Rα + 20nM rapamycin for 48hrs (representative of n = 3 biologically independent samples). (**e**) IEL that were cultured in 100ng/mL IL-15/Rα (+/- 20nM rapamycin) for 96hrs were stained for intracellular GzmA and GzmB (gating strategy shown in Fig. S1a), expression presented as geometric MFI and MFI, respectively (n = 3 biologically independent samples). (**f**) IEL from PIM1/2 dKO mice were isolated and cultured in 100ng/ml IL-15/Rα with (grey) or without (green) 20nM rapamycin for 48hrs, bar graphs show the phospho-S6 (Ser235/236) expression as MFI in IEL subsets (n = 3 biologically independent samples). All error bars are mean ± s.e.m. (e) and (f) analysed by two-way ANOVA with Sidak's multiple comparisons test.

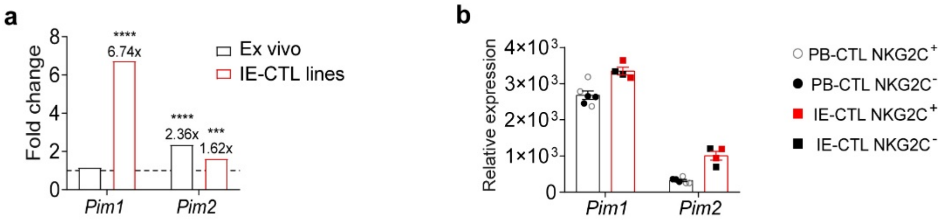

Supplementary Figure 5. *Pim1* and *Pim2* mRNA expression in human IEL derived cell lines.
(**a**) Data derived from GSE120904 and Ciszewski et al, 2020 (ref. 15). *Ex vivo* single-cell suspensions from the epithelial compartment isolated from human duodenal biopsies (ex vivo) or CD8+ intraepithelial cytotoxic T-cell lines (IE-CTL) were stimulated with human recombinant IL-15 for 2 hours and RNA sequencing performed. Bar graphs show the fold change in gene expression (untreated vs IL-15-treated) for *Pim1* and *Pim2* kinases. (**b**) Gene expression profiling by microarray of NKG2C$^+$ and NKG2C$^-$ intraepithelial cytotoxic T cells (IE-CTL) and peripheral blood cytotoxic T cells (PB-CTL) clones generated from four celiac patients obtained from Gene Expression Omnibus dataset GSE4592 (ref. 12). Data show *Pim1* and *Pim2* gene expression in IE-CTL compared to PB-CTL clones.

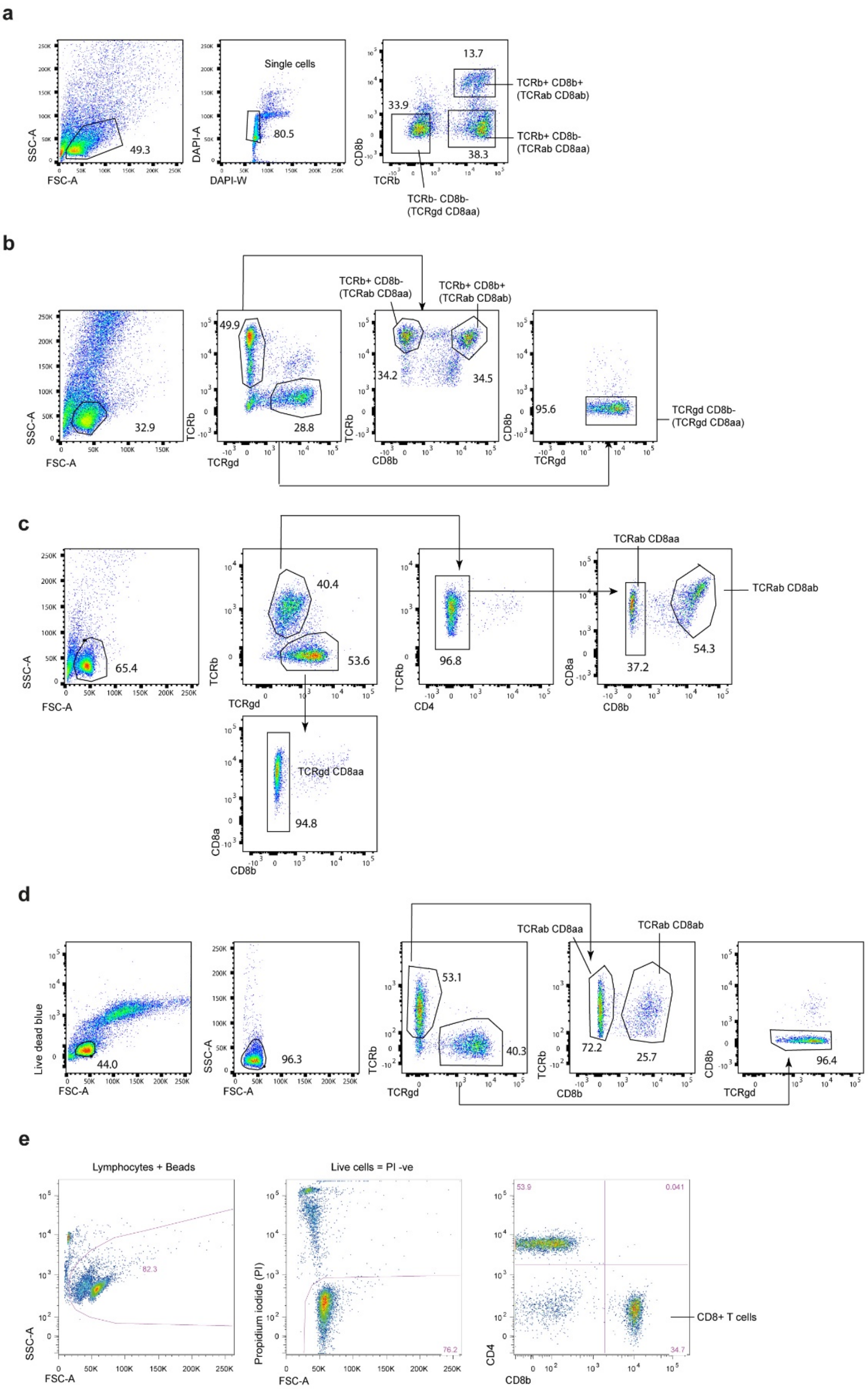

Supplementary Figure 6. Flow Cytometry gating strategies

(**a**) Gating strategy for Fig. 2f (**b**) Gating strategy for Fig. 3d, Fig. S1f and Fig. S4a (**c**) Gating strategy for Fig. 5b, Fig. 7c (right panel) and Fig. S1e (right panel) (**d**) Gating strategy for Fig. S4f (**e**) Gating strategy for Fig. S2f

Supplementary Table 1. Primer list

Table provides a complete list of all primers used, including the names and sequences

Primer	Forward	Reverse
PIM1	5’- CAAACTGTCTCTTCAGAGTG -3’	5’-GTTCCGGATTTCTTCAAAGG-3’
PIM2	5’-ATGTTGACCAAGCCTCTG-3’	5’-CGGGAGATTACTTTGATGG-3’
CD3ε	5’- GAGAGCAGTCTGACAGATAGGAG-3’	5’GAGGCAGGAGAGCAAGGTTC-3’
