# Supplementary material for: IL-15 and PIM kinases direct the metabolic programming of intestinal intraepithelial lymphocytes
Source: Nat Commun. 2021 Jul 13;12:4290. doi: 10.1038/s41467-021-24473-2 (PMC8277781; doi:10.1038/s41467-021-24473-2)
Supplement: Supplementary file 3 — Description of Additional Supplementary Files [file 41467_2021_24473_MOESM3_ESM.pdf]

Description of Additional Supplementary Files

File Name: Supplementary Data 1
Description: Proteins significantly changed at least 2-fold by IL-15/Rα stimulation in each of the three main IEL subsets.

Table contains lists of proteins that were significantly up- or down-regulated >2 fold in each IEL subset (proteins with $P < 0.05$ were deemed significantly changed). Table also shows the overlap of proteins significantly up- and down-regulated in all IEL subsets.

File Name: Supplementary Data 2
Description: Functional annotation clustering of proteins significantly upregulated >2-fold in at least 2 subsets

Table contains a list of proteins significantly ($P < 0.05$) upregulated >2fold in 2 or more IEL subsets based on Venn diagram in Fig. 1e. These proteins were submitted in DAVID bioinformatics analysis as UNIPROT accession IDs to be used for the 'Gene Functional Classification Tool', with low classification stringency setting. All other settings were standard. The Gene functional classification result was exported and is provided in the supplementary table. P-values computed in the functional annotation clustering are derived from an EASE Score (a Modified Fisher Exact P-value)

File Name: Supplementary Data 3
Description: Complete analysed proteomics data for all IEL subsets with intensities, copy numbers and statistics.

This table provides the complete data set including protein intensities, calculated copy numbers and statistical output from the limma DE analysis. Limma uses a two-tailed empirical Bayes moderated *t*-statistics to calculate p-values. P-value adjustment was performed using the BH method, and associated q values were calculated using the Bioconductor package 'qvalue'.

File Name: Supplementary Data 4
Description: Summary table of protein identification in each replicate

Table summarising the number of proteins identified in each replicate of each sample, plus the number of proteins consistently identified in all biological replicates (overlap) across the samples. Only proteins with an intensity value > 0 were counted. The number of those proteins that were identified by MS/MS or by matching between runs are shown in the subsequent columns, for individual samples and for the overlap of biological replicates. Proteins only identified by site, or identified as reverse or contaminants were removed prior to filtering for identification.

File Name: Supplementary Data 5

Description: Antibody List

Table detailing all antibodies used for flow cytometry, immunoblotting and immunohistochemistry. Details of clone, catalogue number and company are provided as well as dilutions used here.
