# Supplementary material for: IL-15 and PIM kinases direct the metabolic programming of intestinal intraepithelial lymphocytes
Source: Nat Commun. 2021 Jul 13;12:4290. doi: 10.1038/s41467-021-24473-2 (PMC8277781; doi:10.1038/s41467-021-24473-2)
Supplement: Supplementary file 9 — Reporting Summary [file 41467_2021_24473_MOESM9_ESM.pdf]

nature research

Corresponding author(s): Mahima Swamy
Last updated by author(s): May 10, 2021

Reporting Summary

Nature Research wishes to improve the reproducibility of the work that we publish. This form provides structure for consistency and transparency in reporting. For further information on Nature Research policies, see Authors & Referees and the Editorial Policy Checklist .

Statistics

For all statistical analyses, confirm that the following items are present in the figure legend, table legend, main text, or Methods section.

n/a	Confirmed	
☐	☒	The exact sample size (*n*) for each experimental group/condition, given as a discrete number and unit of measurement
☐	☒	A statement on whether measurements were taken from distinct samples or whether the same sample was measured repeatedly
☐	☒	The statistical test(s) used AND whether they are one- or two-sided<br>*Only common tests should be described solely by name; describe more complex techniques in the Methods section.*
☐	☒	A description of all covariates tested
☐	☒	A description of any assumptions or corrections, such as tests of normality and adjustment for multiple comparisons
☐	☒	A full description of the statistical parameters including central tendency (e.g. means) or other basic estimates (e.g. regression coefficient) AND variation (e.g. standard deviation) or associated estimates of uncertainty (e.g. confidence intervals)
☐	☒	For null hypothesis testing, the test statistic (e.g. *F*, *t*, *r*) with confidence intervals, effect sizes, degrees of freedom and *P* value noted<br>*Give P values as exact values whenever suitable.*
☒	☐	For Bayesian analysis, information on the choice of priors and Markov chain Monte Carlo settings
☒	☐	For hierarchical and complex designs, identification of the appropriate level for tests and full reporting of outcomes
☐	☒	Estimates of effect sizes (e.g. Cohen's *d*, Pearson's *r*), indicating how they were calculated

Our web collection on statistics for biologists contains articles on many of the points above.

Software and code

Policy information about availability of computer code

Data collection	Proteomics data were acquired on a LTQ-Orbitrap Velos Pro mass spectrometer running Xcalibur software (v. 2.1.0SP1.1160) (Thermo Scientific).<br>Flow cytometry data were acquired on a LSR Fortessa II with DIVA software (version 8.0.1) or a FACSVerse flow cytometer with FACSuite software (version 1.0.5.3841) (BD Biosciences) or a CytoFlex flow cytometer with CytExpert v2.4.0.28 software (Beckman Coulter).<br>High-resolution respirometry experiments were carried out using an Oroboros Oxygraph-2k (Oroboros instruments, Innsbruck, Austria)
Data analysis	Mass spec data was analysed using the MaxQuant software package version 1.6.8.0. MaxQuant-derived data was converted into estimated copy numbers per cell in Excel.<br>For differential expression analyses of proteomic data, P values and fold changes were calculated on log2-normalized copy numbers using the limma package in R, and the Bioconductor package 'qvalue'. For functional annotation analysis of proteins identified, the database for annotation, visualization and integrated discovery (DAVID) Bioinformatics resources 6.862,63 was used (https://david.ncifcrf.gov/tools.jsp).<br>Heat maps were generated using the Morpheus tool from the Broad Institute (https://software.broadinstitute.org/morpheus) or the 'Proteus' package in RStudio (RStudio Team (2016)). Functional interaction networks of proteins were generated using the STRING database, v11.0 (http://string-db.org/). All flow cytometry analysis performed using FlowJo software V10 (TreeStar) and subsequent statistical tests were carried out using GraphPad Prism v.9.

For manuscripts utilizing custom algorithms or software that are central to the research but not yet described in published literature, software must be made available to editors/reviewers. We strongly encourage code deposition in a community repository (e.g. GitHub). See the Nature Research guidelines for submitting code & software for further information.

Data

Policy information about availability of data

All manuscripts must include a data availability statement. This statement should provide the following information, where applicable:

- Accession codes, unique identifiers, or web links for publicly available datasets
- A list of figures that have associated raw data
- A description of any restrictions on data availability

All analysed proteomics data generated during this study are provided in Supplementary Data 3, with further analyses in Supplementary Data 1, 2 and 4. Raw mass spectrometric data files and MaxQuant analyses have been deposited to the ProteomeXchange Consortium via the PRIDE partner repository with the dataset identifier PXD025891. https://www.ebi.ac.uk/pride/archive/projects/PXD025891/
Gene expression data from human samples were obtained from Gene expression Omnibus datasets GSE120904 and GSE4592.
https://www.ncbi.nlm.nih.gov/geo/query/acc.cgi?acc=GSE120904
https://www.ncbi.nlm.nih.gov/geo/query/acc.cgi?acc=GSE4592
A source data file is provided with this manuscript for all figures, excluding data pertaining to; Fig. 1c which is provided in full in supplementary Data 3; data pertaining to Fig. 1d and e are provided in supplementary Data 1; data pertaining to Fig. 1f is provided in supplementary Data 2; and data pertaining to Fig. S1c and d are provided in supplementary Data 3. All other raw data supporting this study can be obtained from the corresponding author upon request.

Field-specific reporting

Please select the one below that is the best fit for your research. If you are not sure, read the appropriate sections before making your selection.

☒ Life sciences ☐ Behavioural & social sciences ☐ Ecological, evolutionary & environmental sciences

For a reference copy of the document with all sections, see nature.com/documents/nr-reporting-summary-flat.pdf

Life sciences study design

All studies must disclose on these points even when the disclosure is negative.

Sample size	For all proteomics experiments 4 biological replicates were generated. Previous published proteomic studies (Howden et al, Nat. Immunol. 2019, Hukelmann et al, Nat. Immunol. 2016) have revealed that at least 3 replicates is sufficient to perform statistical analyses and identify significant changes in protein expression using mass spectrometry, and to increase the power, we have used 4 replicates. In all orthogonal validation experiments, at least 3 biological replicates were performed based on the estimated variability from previous data. For the human tissue immunohistochemistry, n=8 samples were deemed sufficient to identify if the PIM kinases are upregulated in Active CeD versus control samples, based on teh previously published gene expression data from GSE120904 and GSE4592.
Data exclusions	For analyses of the raw proteomic data set, raw data was filtered to remove proteins categorized as “contaminants”, “reverse” and “only identified by site” as pre-established criteria for exclusion in MaxQuant. Following DE analysis, only proteins with > 2 peptides quantified were retained for further functional analyses to reduce the impact of false positive identifications.
Replication	For all experiments at least 3 replicates were generated where possible. All replication attempts were successful
Randomization	Randomization was not possible for this study. Where cells were treated with IL-15, control and treated cells came from the same starting pool of cells.
Blinding	No blinding was done in this study, as it is not possible to perform proteomics analyses blinded. However, the Maxquant analyses of the proteomic were run in an unbiased automated software. For functional validation, it was essentially not possible to perform the experiments blind due to the small sample numbers per experiment.

Reporting for specific materials, systems and methods

We require information from authors about some types of materials, experimental systems and methods used in many studies. Here, indicate whether each material, system or method listed is relevant to your study. If you are not sure if a list item applies to your research, read the appropriate section before selecting a response.

Materials & experimental systems

n/a	Involved in the study	
☐	☒	Antibodies
☐	☒	Eukaryotic cell lines
☒	☐	Palaeontology
☐	☒	Animals and other organisms
☐	☒	Human research participants
☒	☐	Clinical data

Methods

n/a	Involved in the study	
☒	☐	ChIP-seq
☐	☒	Flow cytometry
☒	☐	MRI-based neuroimaging

Antibodies

Antibodies used	Flow Cytometry:<br>FC block (Purified anti-mouse CD16/32 Antibody) (clone 93, BioLegend)<br>CD45 (clone 30.F11, BioLegend)<br>TCRβ (clone H57-597, BioLegend)<br>TCRγδ (clone GL3, BioLegend)<br>TCRγδ (clone GL3, eBioscience)<br>CD4 (clone RM4-5, BioLegend)<br>CD8α (clone 53-6.7, BioLegend)<br>CD8β (clone H35-17.2, eBioscience)<br>CD122 (clone M-b1, eBioscience)<br>NKG2D (clone CX5, BioLegend)<br>CD94 (clone 18d3, BioLegend)<br>JAML (clone HL4E10, BioLegend)<br>CD100 (clone BMA-12, BioLegend)<br>CD223 (LAG3) (clone eBioC9B7W, eBioscience)<br>CD85k (LILRB4) (clone H1.1, BioLegend)<br>CD226 (DNAM-1) (clone 10E5, eBioscience)<br>CD96 (clone 3.3, BioLegend)<br>CD71 (clone RI7217, BioLegend)<br>CD98 (clone RL388, BioLegend)<br>GzmB (clone GB12, eBioscience)<br>GzmA (clone GzA-3G8.5, eBioscience)<br>phospho-STAT5 (Tyr694) (clone C11C5, Cell Signaling Technology)<br>phospho-S6 ribosomal protein (Ser235/236) (clone D57.2.2E, Cell Signaling Technology)<br>anti-rabbit DyLight™649-conjugated donkey secondary Ab (clone Poly4064, BioLegend)<br>Pacific Blue™ Succinimidyl Ester (ThermoFisher Scientific)<br>DyLight™ 800 NHS Ester (ThermoFisher Scientific)<br><br>LIVE/DEAD™ Fixable Blue Dead Cell Stain Kit (ThermoFisher Scientific)<br>DAPI (Invitrogen™)<br><br>Immunoblotting & Imaging:<br>Polyclonal anti-rabbit pan-STAT5 (clone D2O6Y, Cell Signaling Technology)<br>Polyclonal anti-rabbit phospho-STAT5 Tyr694 (cat# 9351, Cell Signaling Technology)<br>Monoclonal anti-mouse PIM1 (clone 12H8, Santa Cruz Biotechnology)<br>Monoclonal anti-mouse PIM2 (clone 1D12, Santa Cruz Biotechnology)<br>Monoclonal anti-rabbit GAPDH (clone 14C10, Cell Signaling Technology)<br>Polyclonal rabbit anti-human CD3 (cat# A0452, Agilent)<br>Anti-mouse IgG, HRP-linked (cat# 7076, Cell Signaling Technology)<br>Anti-rabbit IgG, HRP-linked (cat# 7074, Cell Signaling Technology)
Validation	The commercially available antibodies were validated by the vendors for the species and assay used in our study on their official websites. Antibodies were further titrated to optimise the signal. Further details of antibody validation can be found at:<br>1) https://www.biolegend.com/reproducibility<br>2) https://www.thermofisher.com/ca/en/home/life-science/antibodies/invitrogen-antibody-validation.html<br>3) https://www.cellsignal.co.uk/about-us/cst-antibody-validation-principles<br><br>Validation of the PIM1 and PIM2 antibodies for immunoblotting is shown in Fig. S2.

Eukaryotic cell lines

Policy information about cell lines

Cell line source(s)	K562 expressing luciferase was obtained from Dr. S. Minguet, University of Freiburg.
Authentication	No authentication other than for the expression of luciferase was performed.
Mycoplasma contamination	The cell line was tested and was negative for mycoplasma contamination.

Commonly misidentified lines (See ICLAC register)	No commonly misidentified cell lines were used in the study.

Animals and other organisms

Policy information about studies involving animals; ARRIVE guidelines recommended for reporting animal research

Laboratory animals	Untreated vs IL-15-treated proteomics experiments - C57BL/6J mice, 50-60 days old, male<br>PIM1/2 dKO experiments - PIM1-/- /PIM2-/Y with age and sex-matched non-littermate wild-type controls, 70-300 days old<br>PIM1 sKO proliferation experiments - PIM1-/- mice, with age and sex matched littermate wild-type controls, 70-300 days old<br>PIM2 sKO proliferation experiments - PIM2 -/Y mice, with age and sex matched littermate wild-type controls, 70-80 days old, male<br>GzmA/GzmB dKO killing assay - GzmA-/- / GzmB-/- mice with co-housed age and sex matched C57BL/6 wild-type controls, 80-110 days old. Both male and female mice were used.<br>All other experiments were commercially sourced C57BL/6J mice, 60-170 days old, male<br><br>All C57BL/6 mice were purchased from Charles River, except those used as controls for PIM1/2 dKO, PIM1 sKO, PIM2 sKO and GzmA/GzmB dKO experiments which were bred in the Biological Resource Unit at the University of Dundee.
Wild animals	The study did not involve wild animals.
Field-collected samples	The study did not involve samples collected from the field.
Ethics oversight	Mice were maintained in the Biological Resource Unit at the University of Dundee using procedures that were approved by the University Ethical Review Committee and under the authorisation of the UK Home Office Animals (Scientific Procedures) Act 1986.

Note that full information on the approval of the study protocol must also be provided in the manuscript.

Human research participants

Policy information about studies involving human research participants

Population characteristics	All human tissues were obtained from the NHS Research Scotland Biorepository in Tayside. Formalin-fixed paraffin embedded (FFPE) duodenal biopsies were obtained from patients who had undergone oesophageal-gastro duodenoscopy in which the histological features were subsequently in keeping with coeliac disease (n=8) compared to controls in which features were subsequently within normal limits (n=8). No other clinical information was used or recorded within this study.
Recruitment	All human tissues were obtained with written informed consent from donors under the governance of and with ethical approval from the Tayside Biorepository.
Ethics oversight	The Tayside Biorepository (TBR) holds approval from the Local Research Ethics Committee (LREC) to provide data and samples to researchers who satisfy the TBR Access Committee that their application is both ethically and scientifically appropriate.

Note that full information on the approval of the study protocol must also be provided in the manuscript.

Flow Cytometry

Plots

Confirm that:

- [x] The axis labels state the marker and fluorochrome used (e.g. CD4-FITC).
- [x] The axis scales are clearly visible. Include numbers along axes only for bottom left plot of group (a 'group' is an analysis of identical markers).
- [x] All plots are contour plots with outliers or pseudocolor plots.
- [x] A numerical value for number of cells or percentage (with statistics) is provided.

Methodology

Sample preparation	For all flow cytometry experiments IEL were isolated from murine small intestines, and enriched for CD8α+ IEL using the EasySep™ Mouse CD8a Positive Selection Kit II (STEMCELL Technologies) and cultured with 2, 10 or 100ng/mL or without IL-15/Rα (MO IL-15/IL-15R COMPLEX, Life technologies). Cells were plated at 2x10^5 cells per well in a 96-well dish for staining. FC block was added to each well for 5 minutes before cells were incubated with monoclonal antibodies (mAb) against cell surface markers for 15 minutes at 4˚C. Stained cells were washed with PBS supplemented with 1% FBS and DAPI (1μg/ml).<br><br>For intracellular staining, cells were stained with a fixable live/dead blue dye and then treated with 2% PFA at 37˚C for 10 minutes before cells were washed in permeabilization buffer (eBioscience) and incubated with mAbs specific for murine GzmB (1:200) or GzmA (1:1000) for 1hr at room temperature. For detection of phospho-STAT5 or phospho-S6 ribosomal protein by flow cytometry, cells were fixed with 2% paraformaldehyde (PFA) prior to any surface stains, permeabilised with 90% ice cold

methanol and incubated with phospho-STAT5 (Tyr694) (1:200) or Phospho-S6 Ribosomal Protein (Ser235/236) (1:25 dilution) for 30 minutes at room temperature. Cells were then incubated with an anti-rabbit DyLight™649-conjugated donkey secondary Ab (1:500) for 30 minutes at room temperature.

To measure DNA synthesis or protein synthesis, cells were treated with 10µM baseclick 5-Ethynyl-deoxyuridine (5-EdU) (Sigma) for 2hrs or 20µM O-propargyl-puromycin (OPP) (JenaBioscience) for 15 minutes, respectively. For OPP assays, a negative control was pre-treated with 0.1mg/mL cycloheximide (CHX) solution (Merck) for 30 minutes before adding OPP. Cells were then harvested (~1 million cells per condition), fixed with 4% PFA and permeabilised with 0.5 % triton X-100 before undergoing a copper catalysed click chemistry reaction with Alexa 647-azide (Sigma). Cells were then stained with surface markers as described above and resuspended in FACS buffer (PBS + 1% FBS (+15µg/mL DAPI for cell cycle analysis)) and analysed by flow cytometry to determine the degree of incorporation of EdU or OPP.

For Fluorescence Activated Cell Sorting (FACS) IEL were isolated from the small intestine and further enriched for CD8α+ IEL using an EasySep™ Release PE positive selection kit (STEMCELL Technologies) with a PE-conjugated anti-mouse CD8α antibody as per the manufacturer's instructions. Cells were stained with PerCP eFluor 710 (TCRγδ), APC (TCRβ), PE (CD8α), FITC (CD8β) and PE-Cy7 (CD4) for isolation of pure populations of TCRγδ CD8αα, TCRαβ CD8αα and TCRαβ CD8αβ IEL using an Influx cell sorter (Becton Dickinson).

For Fluorescent Cell Barcoding (FCB) IEL were isolated and enriched for CD8α+ as described above. Cells were resuspended at a concentration of 1million cells/mL and 500µl plated in a 24 flat bottom well plate. Cells were warmed at 37˚C for 30 min before stimulated with different concentrations of IL-15/Rα for 3hrs at 37˚C. After stimulation cells were directly fixed with 500µl PFA 4% 10 min at 37˚C before permeabilisation with 90% ice cold methanol. During methanol permeabilisation, each sample was stained with a mix of various concentrations of amine-reactive fluorescent dyes for 40 min, on ice before quenching with PBS + 0.5%BSA (v:v). Pacific blue dye was used at a concentration of 0µg/ml, 11.1 µg/ml or 100µg/ml and DyLight800 dye at a concentration of 0µg/ml or 25µg/ml. Barcoded samples were then pooled and stained for intracellular p-STAT5 and surface markers as described iabove. Data were acquired using CytoFlex flow cytometer.

Instrument

Flow cytometry data were acquired on a LSR Fortessa II, a FACSVerse flow cytometer. FCB data were acquired on a CytoFlex flow cytometer (Beckman Coulter). Distinct IEL subsets were purified by FACS for proteomic experiments on an Influx cell sorter (Becton Dickinson).

Software

Data was acquired on instruments listed above with FACSDiva or FACSuite software (BD Biosciences) for the LSR Fortessa II and FACSVerse flow cytometers, respectively.

CytExpert for CytoFLEX Acquisition and Analysis Software.

The Influx cell sorter utilised BD FACS software.

All flow cytometry analysis was performed using FlowJo software V10 (TreeStar)

Cell population abundance

We purified ~10million TCRγδ CD8αα, ~5million TCRαβ CD8αα and ~5 million TCRαβ CD8αβ per biological replicate. All 3 populations were sorted to greater than 96% purity according to a post-sort purity check

Gating strategy

The gating strategy used to identify and isolate IEL subpopulations using fluorescence activated cell sorting (FACS). Cells were first gated on by size using forward scatter (FSC) vs side scatter (SSC) to separate out cells of interest from debris/non-lymphoid cells. FSC Width was used to separate out single cells from doublets. Live cells were gated on as those that did not express DAPI, as DAPI only penetrates cells with permeabilised cell membranes such as those of dead cells. Live cells were then separated into three distinct IEL subpopulations based on cell surface marker expression of T cell-associated receptors; TCRγδ, TCRβ, CD4, CD8α and CD8β. The populations sorted were as follows; those that were TCRγδ+ CD8αα+, those that were both TCRαβ+ CD4- and either CD8αα+ or CD8αβ+.

☒ Tick this box to confirm that a figure exemplifying the gating strategy is provided in the Supplementary Information.
